# Hypervirulent *Klebsiella pneumoniae*: an old enemy with a more powerful weapon

**DOI:** 10.1016/j.ebiom.2024.105402

**Published:** 2024-10-09

**Authors:** eBioMedicine

*Klebsiella pneumoniae* widely exists in the environment and in the normal flora of the mouth, skin, and intestines of humans. The classic *K pneumoniae* strains usually affect immune-deficient populations, such as children and people with cancer. However, during evolution, the bacteria acquire new genetic materials to obtain new phenotypes. The emergence of hypervirulent *K pneumoniae*, drug-resistant *K pneumoniae*, or their combination makes them a major public health threat. In the August, 2024, issue of *The Lancet Infectious Diseases*, the Institute for Health Metrics and Evaluation Pathogen Core Group estimated that *K pneumoniae* is the sixth most burdensome pathogen globally, just after pathogens more familiar to the public such as tuberculosis, malaria, and HIV. It causes 31.1 million (95% uncertainty interval [UI] 23.0–41.1) disability-adjusted life-years (DALYs) for people of all ages and 17.2 million DALYs for children younger than 5 years. This heavy burden urges the research community to improve understanding of the virulence and adaptation mechanisms of *K pneumoniae*.

The virulence plasmid and chromosomal acquisition of ICEKp10 might confer the hypervirulence of hypervirulent *K pneumoniae*. Several genes are associated with this phenotype, such as *iucA* (aerobactin synthesis), _*p*_*rmpA* (encodes the regulator for mucoid phenotype), and *peg-344* (putative transporter) on virulence plasmid and chromosomal *IRP2* (yersiniabactin synthesis) and *CLB* (colibactin toxin biosynthesis). However, the relative contributions of these genes to hypervirulence are not well understood. In the September, 2024, issue of *eBioMedicine*, Russo and colleagues constructed isogenic mutants for virulence plasmid, *iucA*, _*p*_*rmpA*, _*p*_*rmpA*_*2*_ (truncated), *IRP2* and *CLBBC* in different clinical isolates of hypervirulent *K pneumoniae* to measure their effects on lethal dose, 50% (LD_50_) in a murine model. They found that virulence plasmid contributed most to the hypervirulent phenotype and curing virulence plasmid increased mean LD_50_ to 7.6 (range 7.0–9.0), a dose similar to classic *K pneumoniae*. Odds ratios in the absence of virulence plasmid–yersiniabactin, virulence plasmid, _p_RmpA–aerobactin, _p_RmpA, aerobactin, and yersiniabactin showed a 250-fold, 67-fold, 20-fold, 16.7-fold, 10-fold, and 2-fold decrease in lethality, respectively. This study strengthens our understanding of hypervirulence mechanisms, suggesting that countermeasures directed at virulence plasmid and yersiniabactin might be most efficient for maximal attenuation of virulence.

The virulence of hypervirulent *K pneumoniae* is associated with overproduction of capsular polysaccharides anchored to the outer membrane of the bacteria, named hypermucoviscosity. A better understanding of the mechanisms of hypermucoviscosity is crucial to develop novel strategies to control hypervirulent *K pneumoniae*. Published in August, 2024, in *Nature Communications*, Wu and colleagues explored how small non-coding RNAs (sRNAs) regulated hypermucoviscosity. They revealed a large RNA regulatory network of hypermucoviscosity, involving 31 sRNAs and 64 mRNAs. They further experimentally validated one sRNA, ArcZ, located in the intergenic region between *ARCB* and *elbB*. ArcZ strongly suppressed the translation of *MLAA* and *FBP*, two hypermucoviscosity-related genes, via direct RNA–RNA base-pairing interactions; overexpression of ArcZ reduced the virulence of *K pneumoniae* in mice. Their study suggests *MLAA* could be a new therapeutic target to treat hypervirulent *K pneumoniae* and ArcZ, or its analogue, might be a drug candidate.

Besides virulence mechanisms, further understanding on the adaptive evolution of hypervirulent *K pneumoniae* in the host is equally important. In the August, 2024, issue of *Proceedings of the National Academy of Sciences of the United States of America*, Song and colleagues reported a phenotypic switch of carbapenem-resistant hypervirulent *K pneumoniae* from a hypermucoviscous to a hypomucoviscous state in the urinary tract of a patient. This switch resulted from decreased expression of *rmpADC*, which is caused by deletion of the upstream insertion sequence ISKpn26. Although this mucoid switch compromised the virulence of hypervirulent *K pneumoniae* in the mouse model, it significantly increased the residence time of the bacteria in the urinary tract by boosting its adhesion to bladder epithelial cells. In addition to mucoid transition, the evolution of virulent plasmids among different strains of carbapenem-resistant *K pneumoniae* also contributes to their adaptation in the host. In a study published in January, 2024, in *Nature Communications*, the authors curated 1219 *K pneumoniae* genome assemblies from isolates collected in 31 regions globally during 2002–19. They found that the sub-lineage ST11-KL64 carbapenem-resistant *K pneumoniae* acquired a pK2044-like virulence plasmid from ST23-KL1 hypervirulent *K pneumoniae*, with a 2698 base-pair region deletion in all ST11-KL64. The deletion involves genes regulating methionine metabolism, enhances antioxidant capacity, and improves the survival of carbapenem-resistant hypervirulent *K pneumoniae* in macrophages. All these mechanistic explorations could facilitate drug discovery and vaccine development.

The emergence of carbapenem-resistant hypervirulent *K pneumoniae* is a severe threat to humans, as carbapenems are usually applied as the last-line antibiotics. Carbapenem-resistant hypervirulent *K pneumoniae* can be derived either from carbapenem-resistant *K pneumoniae* acquiring a plasmid carrying hypervirulence genes or from hypervirulent *K pneumoniae* acquiring a plasmid carrying carbapenem-resistance genes. In the August, 2024, issue of *Drug Resistance Updates*, Liu and colleagues reported that the prevalence of carbapenem-resistant hypervirulent *K pneumoniae* increased during the COVID-19 pandemic, as evidenced by an increase in local endemic clones in different continents. The authors noticed that this process was facilitated by the convergence of plasmids containing both carbapenemase genes and virulence genes. This study indicates that prudent use of antimicrobials and local infection-control policies are essential for preventing and reducing carbapenem-resistant hypervirulent *K pneumoniae*.

Humans have known about *K pneumoniae* for more than a century. However, as it evolves to gain hypervirulence and drug resistance, our battle against this pathogen becomes more challenging and complicated. Improved understanding of its virulence, resistance, and adaptation are crucial for us to develop more efficient treatments. *eBioMedicine*, as a translational publishing platform, welcomes all basic and clinical research on *K pneumoniae*.

